# Identification of a Potent CDK8 Inhibitor Using Structure-Based Virtual Screening

**DOI:** 10.1021/acs.jcim.4c02011

**Published:** 2024-12-31

**Authors:** Tony Eight Lin, Ching-Hsuan Chou, Yi-Wen Wu, Tzu-Ying Sung, Jui-Yi Hsu, Shih-Chung Yen, Jui-Hua Hsieh, Yu-Wei Chang, Shiow-Lin Pan, Wei-Jan Huang, Kai-Cheng Hsu, Chia-Ron Yang

**Affiliations:** 1 Graduate Institute of Cancer Biology and Drug Discovery, College of Medical Science and Technology, Taipei Medical University, Taipei 11031, Taiwan; 2 Ph.D. Program for Cancer Molecular Biology and Drug Discovery, College of Medical Science and Technology, Taipei Medical University, Taipei 11031, Taiwan; 3 School of Pharmacy, College of Medicine, National Taiwan University, Taipei 10051, Taiwan; 4 Warshel Institute for Computational Biology, The Chinese University of Hong Kong (Shenzhen), Shenzhen, Guangdong 518172, People’s Republic of China; 5 Division of Translational Toxicology, National Institute of Environmental Health Sciences, National Institutes of Health, Durham, North Carolina 27709-2233, United States; 6 Department of Traditional Chinese Medicine, Chang Gung Memorial Hospital, Keelung Medical Center, Keelung 20401,Taiwan; 7 Ph.D. Program in Drug Discovery and Development Industry, College of Pharmacy, Taipei Medical University, Taipei 11031, Taiwan; 8 TMU Research Center of Cancer Translational Medicine, Taipei Medical University, Taipei 11031,Taiwan; 9 School of Pharmacy, Taipei Medical University, Taipei 10051, Taiwan; 10 Cancer Center, Wan Fang Hospital, Taipei Medical University, Taipei 11696,Taiwan

## Abstract

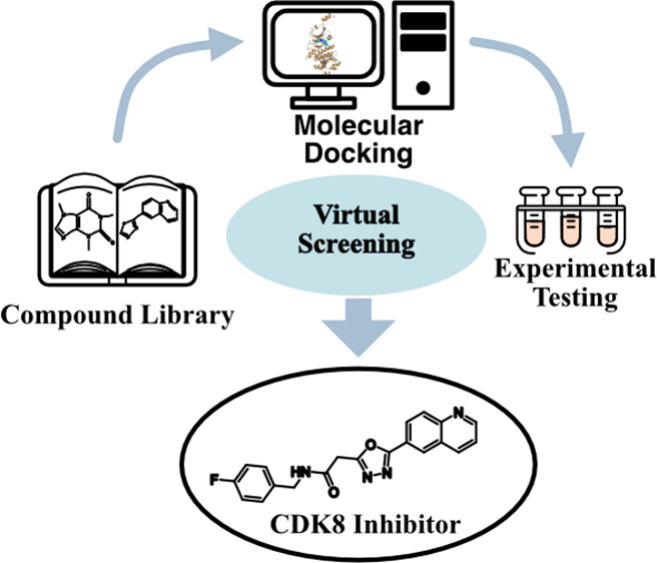

Pulmonary fibrosis is excessive scarring of the lung tissues. Transforming growth factor-beta (TGF-β) has been implicated in pulmonary fibrosis due to its ability to induce the epithelial-to-mesenchymal transition (EMT) and promote epithelial cell migration. Cyclin-dependent kinase 8 (CDK8) can mediate the TGF-β signaling pathways and could function as an alternative therapeutic target for treating pulmonary fibrosis. Here, we performed a structure-based virtual screening campaign to identify CDK8 inhibitors from a library of 1.6 million compounds. The screening process ended with the identification of a novel CDK8 inhibitor, P162-0948 (IC_50_: 50.4 nM). An interaction analysis highlighted important CDK8–ligand interactions that support its binding and inhibitory activity. Testing against a panel of 60 different kinases demonstrated P162-0948 selectivity toward CDK8. Crucially, the inhibitor was found to be structurally novel when compared to known CDK8 inhibitors. Testing in A549 human alveolar epithelial cell lines showed that the P162-0948 can reduce cell migration and protein expression of EMT-related proteins. When P162-0948 was treated in cells at 5 μM, phosphorylation of Smad in the nucleus was reduced, which suggests disruption of the TGF-β/Smad signaling pathway. The identification of P162-0948 shows that it is not only potent, but its structural novelty can inform future design studies for potential therapeutics targeting pulmonary fibrosis.

## Introduction

Chronic inflammation is an underlying issue for a number of diseases, such as arthritis, cardiovascular disease, and cancer.^1^ Excessive inflammation can trigger the wound healing response, leading to overproduction of collagen or extracellular matrix components.^2,3^ Chronic activation of wound healing can result in fibrosis, or scarring, and tissue damage. The lungs are particularly susceptible, with idiopathic pulmonary fibrosis (IPF) effecting 3 million people worldwide.^4^ Current treatment options, such as pirfenidone and nintedanib, may delay the progression of pulmonary fibrosis.^5,6^ However, their long-term use can have detrimental effects, causing patients to reduce or cease treatment.^7,8^ Additional treatments would offer more therapeutic options and potentially improve patient quality of life.

Research into the inflammatory pathways suggests potential avenues for novel therapeutics targeting pulmonary fibrosis.^2,4^ Transforming growth factor-beta (TGF-β) is a crucial cytokine in inflammation and can induce the epithelial-to-mesenchymal transition (EMT), thereby promoting epithelial cell migration.^9,10^ TGF-β works in conjunction with mothers against decapentaplegic homologues (Smad), where activation of the TGF-β/Smad complex leads to its translocation to the nucleus and subsequent binding to DNA.^3,11^ TGF-β has also been implicated in noninflammatory lung fibrogenesis, further positioning it as an important modulator of fibrosis.^12^ Disrupting the TGF-β/Smad axis could be an important strategy for lung fibrosis.

Cyclin-dependent kinase 8 (CDK8) belongs to the serine-threonine class of kinase proteins. Overexpression of CDK8 can result in a number of diseases, such as inflammatory diseases.^13,14^ Crucially, CDK8 can mediate the TGF-β/Smad signaling axis. CDK8 has been reported to form a mediator complex that triggers Smad transcription of EMT genes and collagen.^15,16^ Additionally, inhibition of CDK8 can decrease the signaling of pro-inflammatory cytokines.^11,14,16^ These aspects make CDK8 a promising target for therapeutics targeting fibrosis. However, small molecules targeting CDK8 can present many issues, such as selectivity and toxicity.^17^ Identifying novel CDK8 inhibitors would help inform future drug designs, which would give rise to CDK8 inhibitors with greater selectivity and potency.

**Figure 1:**
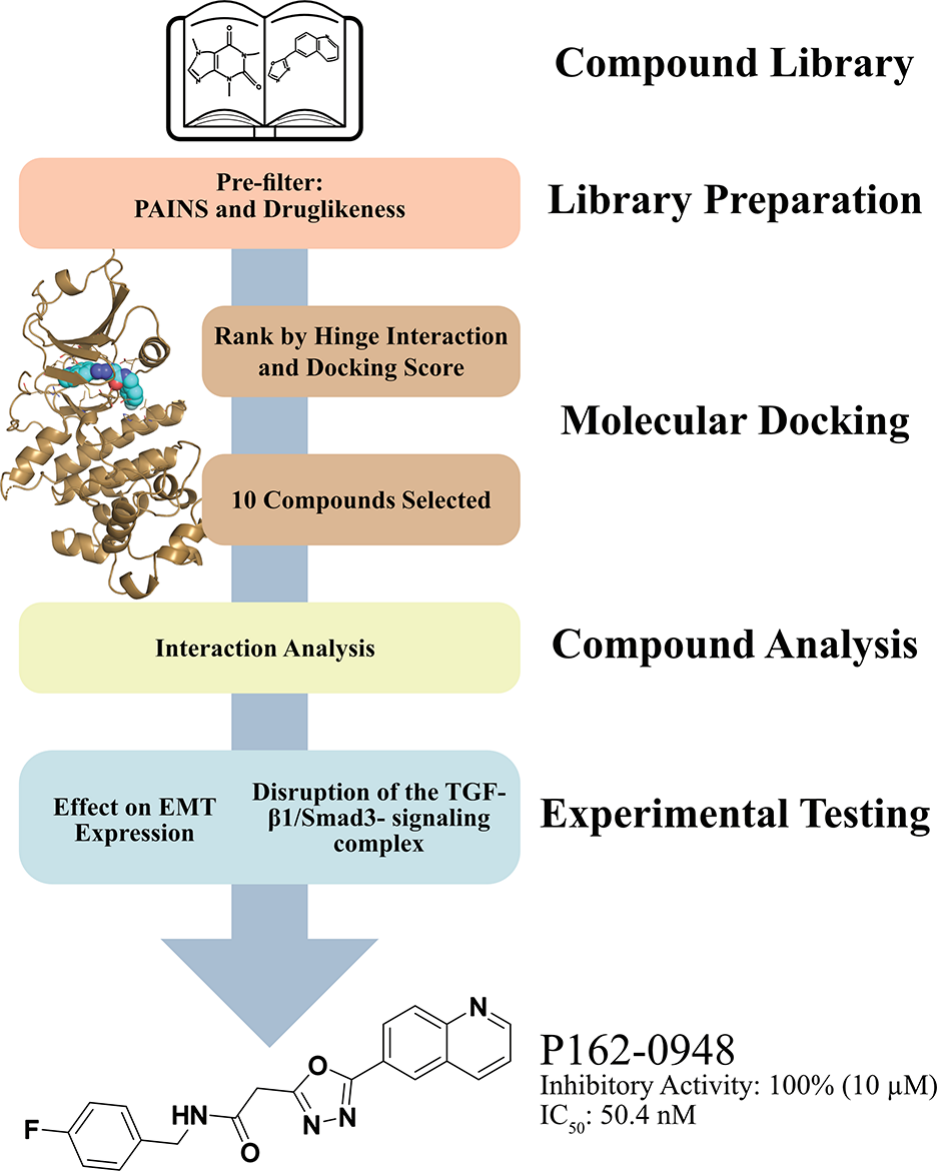
SBVS workflow. The screening library was preprocessed to enrich molecules with potential druglike features. Molecules were molecularly docked and filtered for hinge interactions and ranked by docking scores. Potential inhibitors were selected for compound analysis and experimental testing. The process led to the identification of P162-0948.

Here, we report a structure-based virtual screening (SBVS) campaign to identify CDK8 inhibitors (Figure 1). A commercial screening library containing 1.6 million molecules was molecularly docked into the CDK8 binding site, leading to the identification of a potent CDK8 inhibitor. Structural analysis identified key protein–ligand interactions with preferences for Type II CDK8 inhibitors. Further, kinase assays showed the compounds to have high selectivity for CDK8 inhibition. When testing the candidate compound in A549 cells, we found that it modulated the TGF-β/Smad signaling and reduced EMT-associated protein expression. The identification of these molecules provides researchers additional avenues to explore for future CDK8 inhibitor designs.

## Method

### Molecular Docking

Molecular docking was performed using the Schrödinger Maestro software suite.^18^ The crystal structure of CDK8 (PDB ID: 4F6U) was obtained from the Protein Data Bank.^19^ The protein structure was prepared using the Protein Preparation module in Maestro. This step involved removing water molecules and adding hydrogen atoms and charges to the protein structure. The docking grid was generated using the Receptor Grid Generation module and centered on the cocrystal ligand (Ligand ID: 0SR). Molecules were prepared by generating their 3D structures and protonated using the LigPrep module. Molecular docking was performed using Glide^20^ in the Ligand Docking module.

### Structure-Based Virtual Screening

The SBVS protocol used in this study consisted of several filtering steps. The commercial small molecule library (∼1.6 million) from ChemDiv was utilized as the screening library. The screening library was prefiltered using pan-assay interference (PAINS) and Lipinski and Veber Rules.^21−23^ Next, remaining compounds were molecularly docked as described above. The docking poses were screened for interactions with the CDK8 hinge residues D98, Y99, and A100.^24^ The remaining molecules were then reranked based on their docking scores. The final selection was then made by inspecting the binding poses of the top 100 scoring molecules and considering stock availability.

### Interaction Analysis

Protein–ligand interactions were identified using Pipeline Pilot.^25^ Interactions included hydrogen-bonding and hydrophobic interactions. The protein-compound complexes were visualized using the PyMOL.^26^ Additional information regarding structures in the kinase binding site was obtained from the KinFragLig^27^ library.

### Kinase Assay

Cell-free *in vitro* assays were performed using the SelectScreen kinase profiling service by ThermoFisher Scientific (www.thermofisher.com/selectscreen). In brief, CDK8 used the LanthaScreen EU Kinase Binding Assay. A solution containing 50 mM HEPES pH 7.5, 0.01% BRIJ-35, 10 nM MgCl_2_, 1 mM EGTA, 5 nM CDK8, 2 nM EU-anti-GST antibody, and 10 nM Tracer 236 was mixed. Binding of the antibody and fluorescence tracer to the kinase results in high FRET fluorescence. If the compound binds to the kinase, the tracer is “blocked” from binding to the kinase, resulting in low FRET fluorescence. A ratio of fluorescence emissions between the control and tested molecule is then calculated. Each assay was performed in duplicate. Quality control standards were monitored by ThermoFisher. Selectivity assays were performed across 60 different protein kinases using either the Z’-Lyte or LanthaScreen assays. IC_50_ values and the dose–response curve were calculated using the Python package py50 (https://github.com/tlint101/py50.git).

### Chemical Space Analysis

Data was processed in Python 3.11. Molecules from ChEMBL Version 34 (https://www.ebi.ac.uk/chembl/) were downloaded using the chembl-downloader package.^28^ Molecules with reported CDK8 inhibitory activity labels (*K*_i_, *K*_d_, IC_50_, or EC_50_) ≤ 1,000 nM were considered active. From this set, molecules were clustered, and 30 diverse structures were randomly selected using the Datamol package.^29^ Molecules were converted into 2,048-bit Extended Connectivity Fingerprints (ECFP4), and their Tanimoto similarity scores were calculated using Datamol. Visualization of the CDK8 chemical space was calculated using t-SNE. The molecular fingerprints of molecules were reduced to two components for plotting. Both the matrix cluster map and the scatterplot were generated using the Seaborn package.^30^

### Cell Culture

Human alveolar epithelial A549 cells were obtained from the Bioresource Collection and Research Center in Hsinchu City, Taiwan. Cells were maintained in Dulbecco’s Modified Eagle Medium (DMEM) provided by Invitrogen Life Technologies, Carlsbad, CA, USA. The medium was supplemented with 10% fetal bovine serum (Invitrogen Life Technologies, Carlsbad, CA, USA), along with penicillin (100 U/mL) and streptomycin (100 μg/mL) from Biological Industries, Kibbutz Beit Haemek, Israel. Cells were cultured at 37 °C in a humidified atmosphere containing 5% CO_2_.

### Cell Cytotoxicity Assay

Cytotoxicity was assessed using the MTT assay. Cells (1 × 10^4^) were seeded in 1 mL of medium in 96-well plates and treated with a control vehicle or a vehicle containing the test compound for 12, 24, or 48 h. After treatment, 1 mg/mL MTT was added, and the plates were incubated at 37 °C for 2 h. Cells were then lysed with 10% SDS containing 0.01 M HCl, and absorbance was measured at 570 nm using a microplate reader.

### Immunoblot Analyses

Cells (1 × 10^6^) were lysed in a buffer containing 20 mM HEPES (pH 7.4), 50 mM β-glycerophosphate, 2 mM EGTA, 0.1% Triton X-100, 10% glycerol, 1 μg/mL leupeptin, 1 mM DTT, 1 mM phenylmethylsulfonyl fluoride, 5 μg/mL aprotinin, and 1 mM sodium orthovanadate. After a 10 min incubation at 4 °C, the cells were scraped, followed by an additional 10 min incubation on ice. The samples were then centrifuged at 17,000*g* for 30 min at 4 °C. For analysis, 20 μg of protein from each sample was separated using SDS-PAGE and transferred onto a polyvinylidene difluoride (PVDF). The membrane was then blocked with a solution of 5% BSA in TBST (Tris-buffered saline with 0.1% Tween-20) for 30 min at ambient temperature. Immunoblotting involved an overnight incubation at 4 °C with primary antibodies diluted in TBST, followed by a secondary antibody incubation at room temperature for 1 h. Detection was executed using an ECL reagent (GE Healthcare Corp., Buckinghamshire, UK) and visualized on a photographic film.

### Wound Healing Assay

Cells were seeded in 6-well plates. When cells reached 90% confluence, a scratch was made in the cell monolayer using a sterile 10 μL pipet tip, followed by a medium wash to eliminate detached cells. The cells were then treated with or without test compounds for 24 h. Wound closure was documented through photographs, and the extent of closure was analyzed using ImageJ software (National Institutes of Health, Bethesda, MD, USA).

### Cellular Fractionation Procedure

The cytosolic and nuclear components were separated by a Nuclear/Cytosol Fractionation Kit (Biovision, Inc., Milpitas, CA, USA). Cells were harvested and centrifuged at 600*g* for 5 min. The supernatants were discarded, and the pellets were resuspended in cytosol extraction buffer-A. The mixture was vortexed vigorously for 15 s and then incubated on ice for 10 min. Cytosol extraction buffer-B was subsequently added, vortexed for 5 s, incubated on ice for 1 min, and centrifuged at 20,000*g* to separate the cytosolic fraction. The remaining pellet was then resuspended in a nuclear extraction buffer, vortexed for 15 s, and placed on ice for 10 min. This step was repeated four times. Finally, the samples were centrifuged at 20,000*g* to isolate the nuclear fraction.

### Statistical Analysis

The data are presented as mean ± SEM. A one-way ANOVA was conducted to assess the differences among groups. When ANOVA results were significant, the Tukey’s post hoc test was used to determine which groups exhibited statistically significant differences. Statistical significance was defined as a *p*-value less than 0.05.

## Results

### Structure-Based Virtual Screening Campaign

In this study, an SBVS campaign was carried out to identify potential CDK8 inhibitors (Figure 1). The screening library (1.6 million molecules) was obtained from the commercial vendor ChemDiv. Several filtering steps were taken to prepare the screening library. This included removal of molecules containing PAINS substructures or those that violated Lipinski and Veber Rules.^21−23^ The remaining molecules were molecularly docked into the CDK8 binding site.

Selection of crystal structures included several stages. All CDK8 cocrystal structures were obtained and sorted by their resolution and conformation of the DMG loop (Figure S1A). The position of the DMG loop can dictate access to a back hydrophobic pocket, and a preference was given for CDK8 structures with the DMG-out conformation.^27,32,33^ The cocrystal ligands from the top structures were extracted and then docked into each candidate structure (Figure S1B). The structures were then assessed for docking performance by mixing the cocrystal ligands and 990 molecules randomly selected from the Available Chemical Directory (ACD)^31^ (Figure S1C). When ranking the results, both 4F6U and 4F6W produced favorable docking performance; however, 4F6U was selected due to its resolution (2.1 Å). Additionally, the redocked pose of 4F6U showed the most favorable reproduction to the cocrystal ligand (Figure S1D).

Finally, the prepared screening library was docked into the 4F6U CDK8 binding site. The docking poses were filtered for the presence of hydrogen bonds to CDK8 hinge residues D98, Y99, and A100.^24^ The remaining compounds were ranked by their respective docking scores. Visual inspection was then carried out on the top 100 scoring compounds, and selections were made based on availability. This process led to 10 molecules selected for enzyme-based validation (Table 1).

**Table 1:**
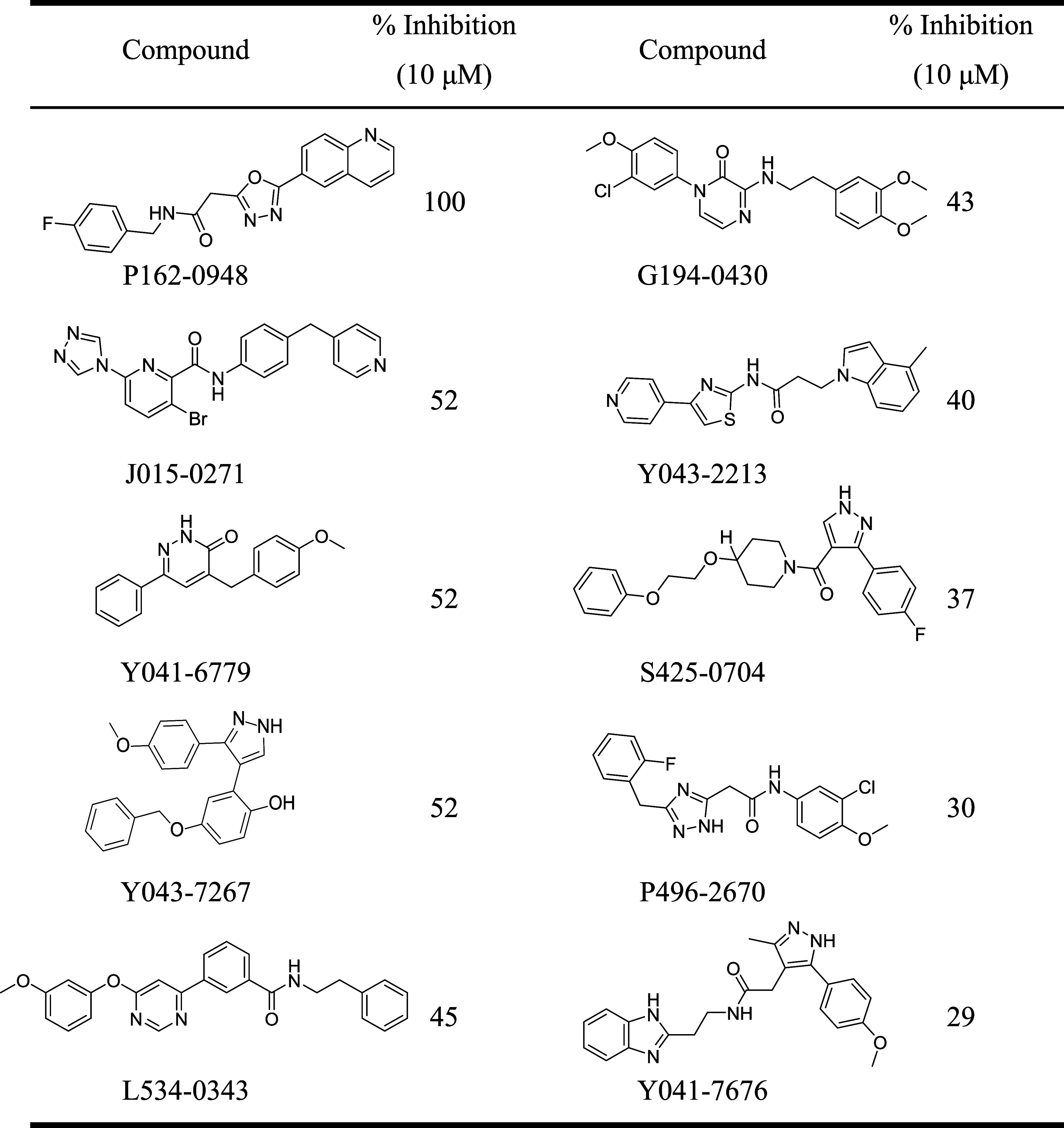
Selected Compounds and Their Inhibitory Activity

### Validation of Selected Molecules

**Figure 2:**
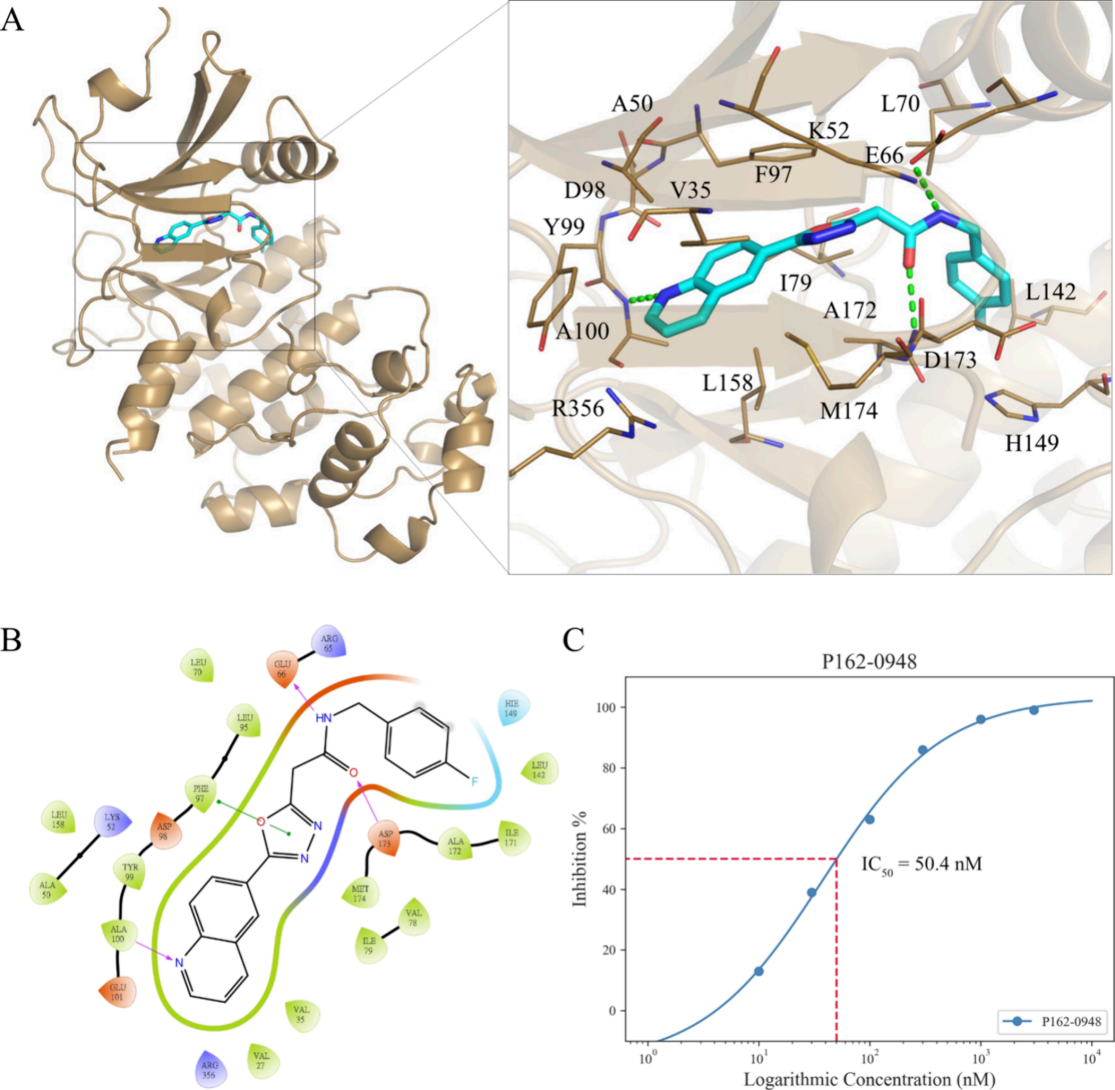
P162-0948 shows favorable interactions with CDK8. (A) Molecular docking pose of P162-0948 (blue) in CDK8 (brown). The inset displays the 3D spatial orientation of the CDK8 binding site. (B) The 2D interaction chart highlights the hydrogen bonds and the hydrophobic interactions. Hydrogen bonds are highlighted with green dashed lines in the 3D image. Hydrogen bonds in the 2D image are represented as purple arrows. The π-stacking interaction is highlighted with the green arrow. Hydrophobic pockets are highlighted with the green line. Binding site residues are labeled as shown in corresponding 3D and 2D images. (C) Dose–response curve for P162-0948. The red box highlights the absolute IC_50_ value.

The 10 selected molecules were tested for CDK8 inhibitory activity at 10 μM. Of the molecules tested, four were found to inhibit CDK8 activity greater than 50%. Importantly, compound P162-0948 exhibited 100% inhibition of CDK8 activity (Table 1). Analysis of the P162-0948 docking pose showed several favorable protein–ligand interactions within the CDK8 binding site (Figure 2A,B). P162-0948 contains a quinoline scaffold that occupies the adenine pocket. This pocket is positioned near the CDK8 hinge residues and the nitrogen on the scaffold functions as a hydrogen acceptor for the backbone of hinge residue A100. Two hydrogen bonds are also observed at the opposite end of the molecule, with the carbonyl oxygen and nitrogen acting as a hydrogen acceptor and donor to residues D173 and E66, respectively. Additionally, hydrophobic interactions are observed at the adenine pocket consisting of residues A50, K52, and Y99. An aromatic π-stacking interaction was also found between residue F97 and the central 1,3,4-oxadiazole of P162-0948. Further testing revealed that P162-0942 has an IC_50_ value of 50.4 nM (Figure 2C). Together, the favorable interactions help facilitate the potent IC50 value observed by the CDK8 inhibitor P162-0948.

### Interaction Analysis and Structural Characteristics of P162-0948

To better determine the interactions that may contribute to potency, an interaction analysis was performed to compare P162-0948 with the selected compounds. Kinase inhibitors can be organized into four distinct classes based on their binding conformation.^32^ The CDK8 structure (PDB ID: 4F6U) used in this study is reported to have a DMG-out conformation, providing access to a hydrophobic back pocket.^27,32,33^ Type II CDK8 inhibitors also occupy the hydrophobic back pocket (Figure S2). This could indicate that molecules selected from the above screening protocol could contain structural features favoring Type II kinase inhibitors. In particular, P162-0948 can occupy the hydrophobic back pocket that is available due to the DMG-out conformation (Figure 2). Comparing its conformation to crystal structures of CDK8 inhibitors with the DMG-out conformation reveals a similar occupation of the hydrophobic back pocket for ligands NZ8 and OSR.^34,35^ Along with P162-0948, known Type II kinase inhibitors can generate interactions with residues in the adenine pocket (red), gate area (yellow), and a hydrophobic back pocket (blue) (Figure S2A,B).

**Figure 3:**
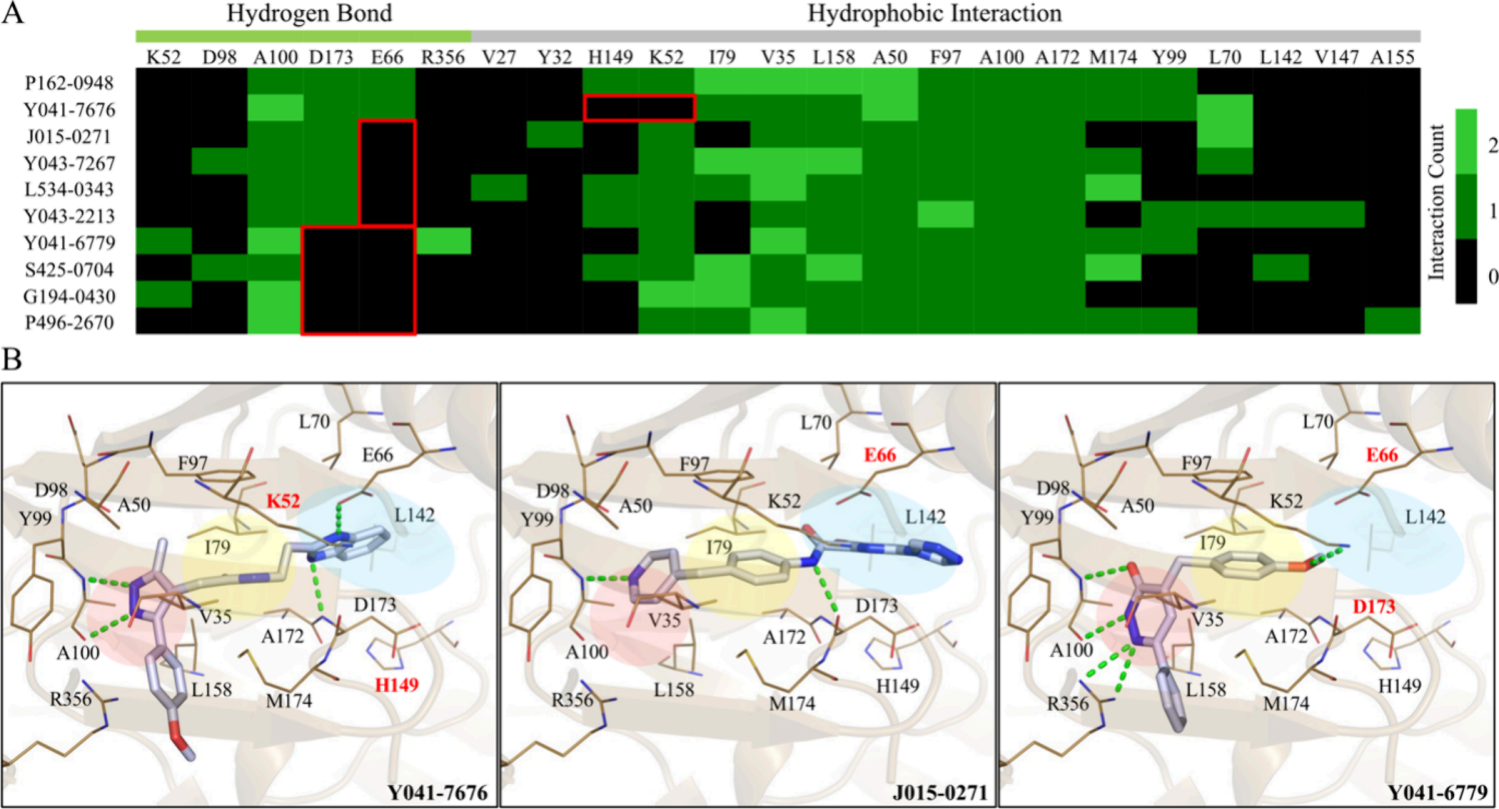
Interaction analysis of selected compounds. (A) The inactive compounds lack several hydrogen bonds and hydrophobic interactions that may reduce their potencies. Red boxes highlight differing interactions compared to P162-0948. (B) The docking poses of the molecules show that the compounds have unfavorable positioning within the CDK8 binding site. This includes the adenine pocket (red), gate area (yellow), and hydrophobic back pocket (blue). Hydrogen bonds are denoted as green lines.

In contrast, the selected molecules with low CDK8 inhibitory activity occupy these positions in varying degrees, which may reduce their binding affinity to the CDK8 DMG-out conformational form (Figure 3A). All molecules selected from the SBVS contain at least one hydrogen bond to the hinge residue. While hydrogen bonds to the hinge region are important, most small-molecule kinase inhibitors in the ATP binding site form at least one hydrogen bonds and a high number of hydrogen bonds may not necessarily indicate potency.^24^ Many of the selected molecules lack hydrogen bonds to D173 of the DMG loop and to residue E66 in the hydrophobic back pocket (Figure 3). Both residues are highly conserved and serve important functions, with residue E66 involved in forming a salt bridge with K52 to stabilize the active CDK8 conformation.^13,34,36^ Additionally, the hydrogen bond found between K52 and P162-0948 would suggest a possible shift of positioning for K52, thereby disrupting the formation of the salt bridge.^13^ Occupation of the hydrophobic back pocket is a common characteristic of Type II inhibitors.^32,37^ The docking poses of the molecules show unfavorable positioning for the CDK8 binding site, with some molecules unable to effectively occupy the back hydrophobic pocket (Figure 3B). This is in contrast to known CDK8 Type II inhibitors, such as ligands NZ8 and OSR. Both contain large moieties that occupy the hydrophobic back pocket (Figure S2). Ligand NZ8 has been reported to have an IC_50_ value of 8.25 nM. In contrast, the identified inhibitor P162-0948 has an IC_50_ value of 50.4 nM (Figure 2C). Further optimization studies to P162-0948 could target modifications for the hydrophobic back pocket.

### Selectivity of P162-0948

**Figure 4:**
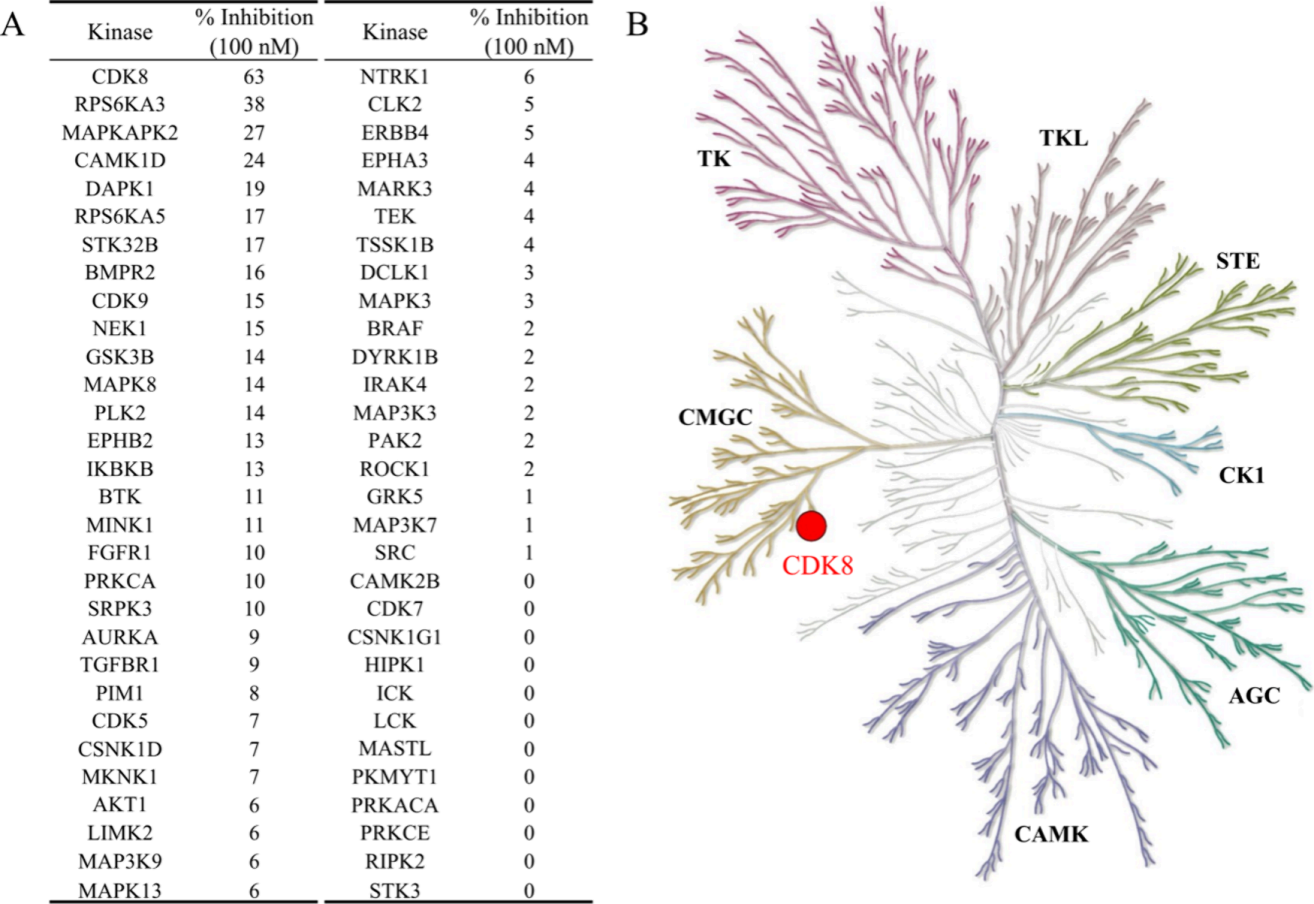
Selectivity assay of P162-0948. (A) A panel of 60 different kinases was selected for testing. P162-0948 was tested at 100 nM. (B) The selected kinases encompass the different kinase families. P162-0948 showed the greatest inhibitory activity for CDK8.

Protein kinases share a similar ATP binding site. As a result, small-molecule kinase inhibitors can target a number of kinase inhibitors. To determine the kinome inhibitory profile of P162-0948, a test targeting 60 different kinases was performed. The selected kinases are spread across the kinome. When P162-0948 was tested at 100 nM, it displayed the greatest inhibitory activity toward CDK8, with an inhibitory activity of 63% (Figure 4A). No other kinase tested produced inhibitory activity greater than 50%. CDK9 belongs to the cyclin-dependent kinase family and is structurally closest to CDK8 (Figure 4B). However, it produced a paltry inhibitory activity of 15%. These results indicate that P162-0948 is selective toward CDK8.

### P162-0948 Is a Structurally Novel CDK8 Inhibitor

**Figure 5:**
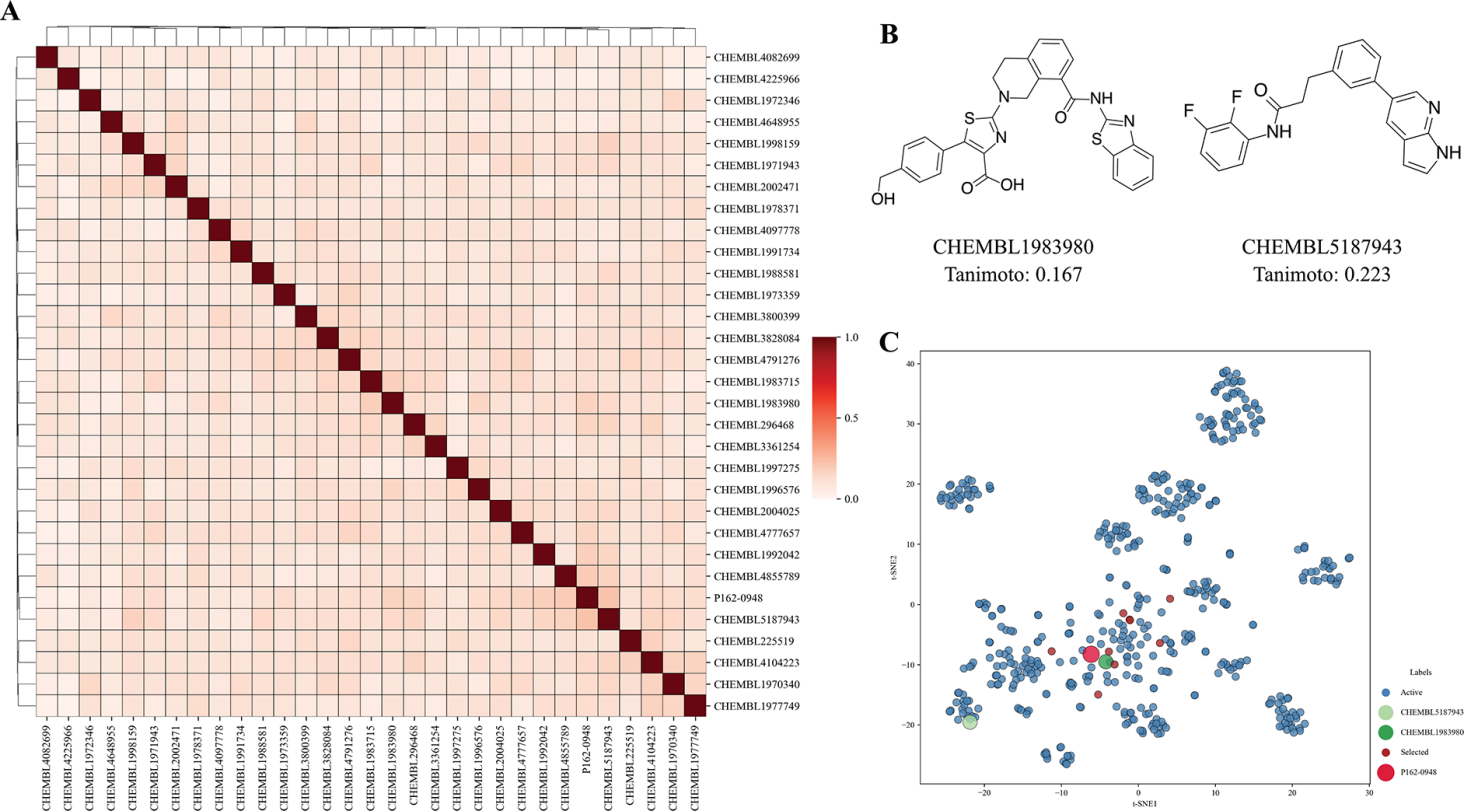
Similarity analysis of CDK8 inhibitors. Known CDK8 inhibitors were analyzed using Tanimoto calculations and t-SNE. (A) A matrix of Tanimoto scores was calculated from 30 diverse CDK8 inhibitors. (B) Examples of CDK8 inhibitors and their calculated Tanimoto scores show little similarity to P162-0948. (C) Visualization of the CDK8 chemical space shows that selected molecules in the study are unrelated to known CDK8 inhibitors.

Common issues plaguing kinase inhibitors include selectivity, potency, toxicity, and the development of resistance.^32,38^ Identifying structurally novel inhibitors can inform future drug design opportunities. With that goal, P162-0948 was structurally compared to known CDK8 inhibitors obtained from the ChEMBL database. A set of 30 diverse molecules was randomly selected and similarity scores between the compounds were calculated using Tanimoto.^39^ When compared to P162-0948, the most similar CDK8 inhibitor, CHEMBL5187943, produced a Tanimoto score of 0.223, indicating little similarity among the molecules (Figure 5A,B). In addition, the *t*-distributed stochastic neighbor embedding (t-SNE) approach can visualize high-dimensional data sets, such as molecular fingerprints.^40,41^ Interestingly, the molecules selected from the virtual screen did not occupy distinct clusters and are structurally distinct from known CDK8 inhibitors (Figure 5C). In contrast, CHEMBL5187943 can be found in a distinct cluster. The CDK8 inhibitor CHEMBL1983980, which is also found in the similarity matrix, is also situated near P162-0948 in the t-SNE plot (Figure 5B,C). Nevertheless, the structural analysis suggests that P162-0948 is structurally unique to known CDK8 inhibitors.

### P162-0948 Modulates TGF-β1 Signaling through CDK8 Inhibition

**Figure 6:**
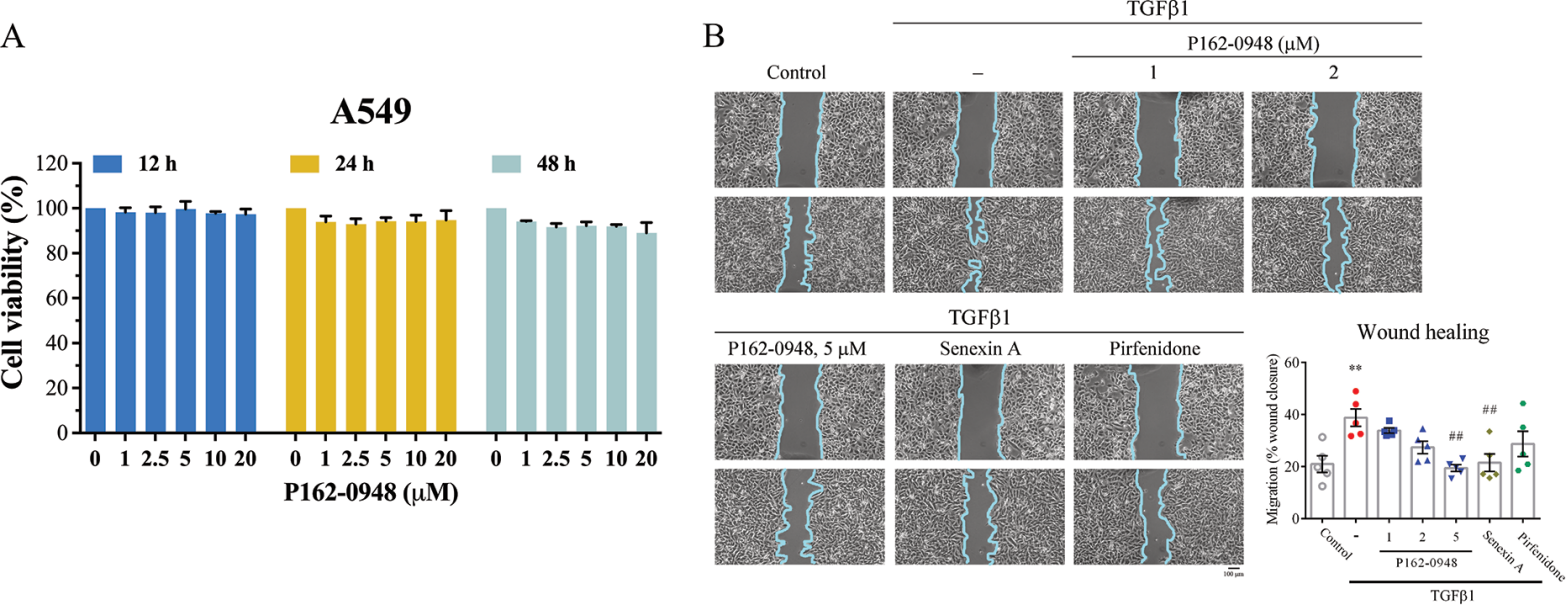
Assessment of cytotoxicity and migration inhibition by P162-0948 in A549 cells. (A) The MTT assay was employed to measure cell viability in A549 cells treated with the indicated concentrations of P162-0948 for 12, 24, or 48 h. (B) A549 cells were cultured in six-well plates until they reached 90% confluency. A pipet tip was used to create a scratch, and images were taken immediately. Cells were then incubated with or without P162-0948 (1, 2, and 5 μM), senexin A (5 μM), or pirfenidone (1 mM) for 1 h, followed by treatment with TGF-β1 (10 ng/mL), and allowed to migrate into the wound area for 24 h. The cells were photographed again; the cyan solid line marks the boundary between the cell-occupied and wound areas (40× magnification). Cell migration into the wound was quantified using ImageJ software. The results are shown as the mean ± SEM from three independent experiments. **p < 0.01 compared to the control group; ##*p* < 0.01 compared to the TGF-β1-treated group.

We next evaluated the effects of P162-0948 on the cell viability of A549 human alveolar epithelial cells using the MTT assay. Administering P162-0948 to A549 cells for 12, 24, and 48 h did not cause significant cytotoxicity. The cellular IC_50_ values of P162-0948 at these time points were greater than 20 μM (Figure 6A). Subsequent experiments would set the P162-0948 concentration to be less than 20 μM to ensure that the compound does not cause cytotoxicity. Next, we determined whether CDK8 inhibitor P162-0948 can impede TGF-β1-induced cell migration.^10^ A549 alveolar epithelial cells were treated with TGF-β1; after 24 h, the cells exhibited a sharper morphology and significant cell migration (Figure 6B). P162-0948 exhibited concentration-dependent inhibition of migration in TGF-β1 treated cells. For comparison, senexin A (a known CDK8 inhibitor) and pirfenidone were employed. We found that 5 μM P162-0948 was more effective than senexin A at 5 μM and pirfenidone at 1 mM (Figure 5B). Thus, P162-0948 can disrupt TGF-β1-induced cell migration.

### P162-0948 Reduces TGF-β1-Mediated EMT Protein Expression

**Figure 7:**
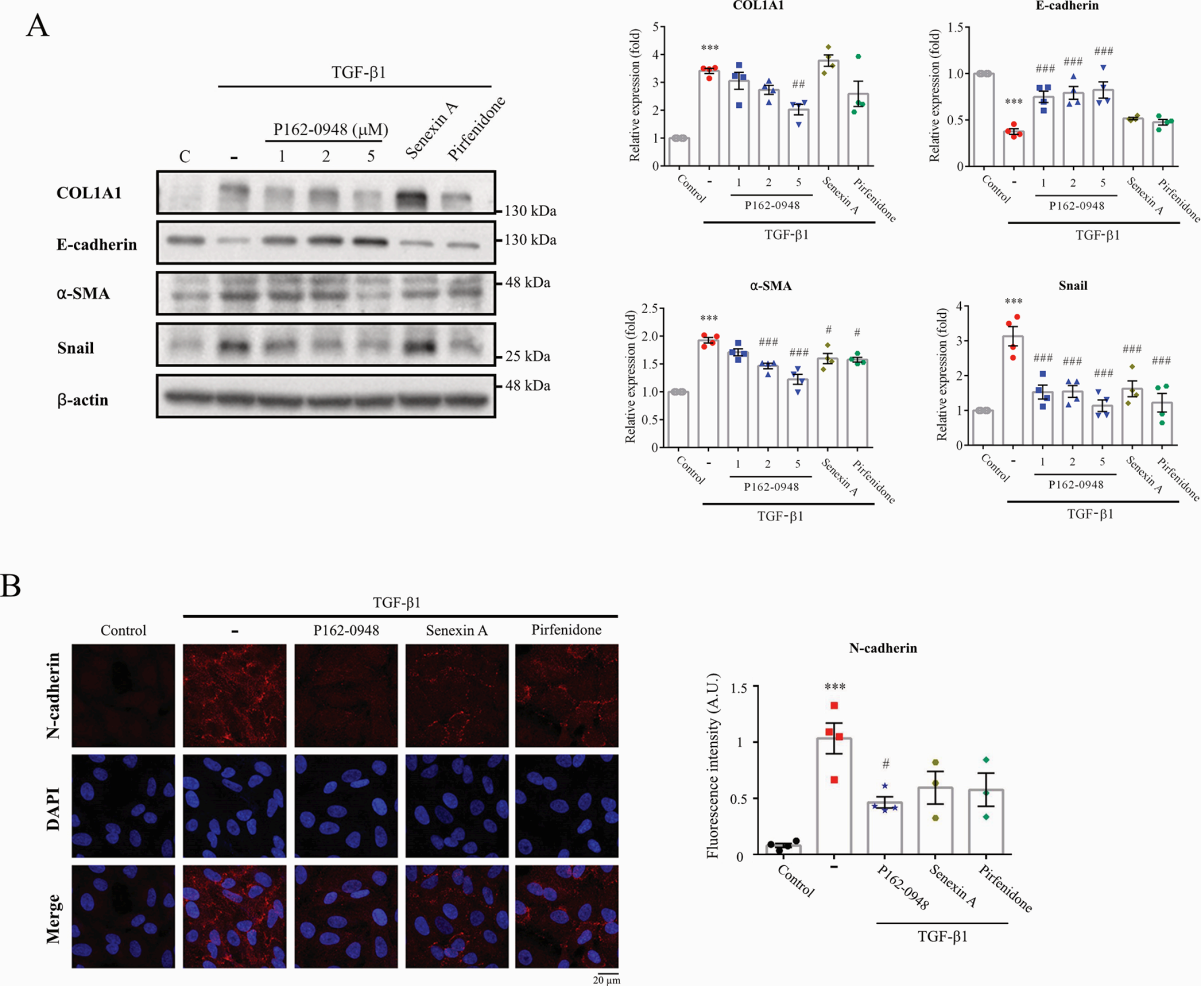
P162-0948 suppressed the expression of EMT proteins in human alveolar epithelial cells. (A) A549 cells were treated with P162-0948 (1, 2, and 5 μM), senexin A (5 μM), or pirfenidone (1 mM) for 1 h, followed by TGF-β1 stimulation (10 ng/mL) for an additional 1 h. Western blot analysis was performed on cell lysates. (B) A549 cells were exposed to P162-0948 (5 μM), senexin A (5 μM), or pirfenidone (1 mM) along with TGF-β1 (10 ng/mL) for 24 h. Immunofluorescence images were captured using a Zeiss LSM880 confocal microscope at 400× magnification (Scale bar: 20 μm). Results are shown as the mean ± SEM from three independent experiments. ***p < 0.001 compared to the control group; #*p* < 0.05, ##*p* < 0.01, and ###*p* < 0.001 compared to the TGF-β1-treated group.

Next, we examined if P162-0948 could suppress TGF-β1-induced EMT protein expression. Previous studies have shown that increased TGF-β1 signaling can stimulate EMT, leading to fibrosis.^9,10^ Cells treated with TGF-β1 notably increased the levels of myofibroblast markers, such as collagen I (COL1A1), α-smooth muscle actin (α-SMA), and Snail, in A549 human alveolar epithelial cells, while reducing the epithelial marker E-cadherin (Figure 7A). These observations indicate the triggering of EMT protein expression. A549 cells treated with P162-0948 and TGF-β1 showed a reduction in EMT protein expressions in a concentration-dependent manner (Figure 7). N-Cadherin can be upregulated by increased TGF-β1 signaling.^42^ Immunofluorescence analysis demonstrated that A549 cells treated with P162-0948 significantly reduced TGF-β1-induced N-cadherin expression (Figure 7B). Senexin A and pirfenidone exhibited less pronounced effects on TGF-β1-induced EMT-related protein levels when compared to P162-0948. These results further suggest that P162-0948 can interfere with TGF-β1-induced EMT signaling.

### P162-0948 Disrupts the TGF-β1/Smad3 Signaling Pathway

**Figure 8:**
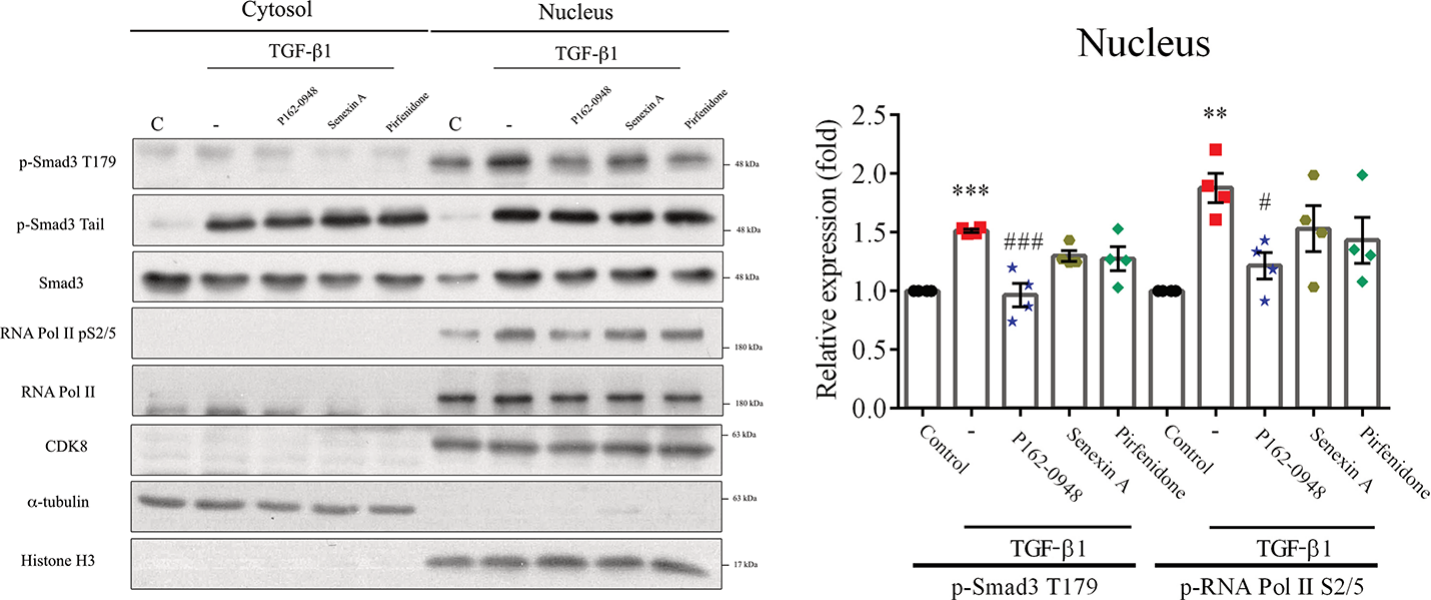
P162-0948 inhibited TGF-β1-induced p-Smad3 T179 and RNA polymerase II pS2/S5. A549 cells were treated with P162-0948 (5 μM), senexin A (5 μM), or pirfenidone (1 mM) for 1 h and then stimulated with TGF-β1 (10 ng/mL) for 3 h. Proteins from cytoplasmic and nuclear fractions were analyzed by Western blot using the specified antibodies. α-Tubulin and histone H3 were used as loading controls for the cytoplasmic and nuclear fractions, respectively. The results are presented as mean ± SEM from three independent experiments. **p < 0.01 and ***p < 0.001 compared to the control group; #*p* < 0.05 and ###*p* < 0.001 compared to the TGF-β1-treated group.

Next, we investigated the intracellular distribution of total and phosphorylated Smad3. Previous studies have shown that CDK8 forms a mediator complex that regulates transcription.^11,43^ CDK8 can also phosphorylate Smad, thereby impacting the TGF-β1/Smad3 signaling pathway.^9,11^ Cells were fractionated into cytoplasmic and nuclear components, using α-tubulin and histone H3 as markers. Under basal conditions, phosphorylated Smad3 at T179 was slightly elevated in the nucleus when compared to its levels in the cytoplasm. However, TGF-β1 treatment significantly elevated nuclear p-Smad3 T179 and RNA polymerase II pSer2/5 levels when compared to the control group (Figure 8). These results suggest that TGF-β1 facilitates the nuclear movement of Smad3, its phosphorylation at threonine 179, and the subsequent activation of RNA polymerase II in A549 cells. Treatment with P162-0948 significantly decreased the phosphorylation levels of Smad3 at T179 and RNA polymerase II at pSer2/5. Reference compounds senexin A and pirfenidone were less effective in reducing these phosphorylation events (Figure 8). Collectively, these findings indicate that P162-0948 is a potent CDK8 inhibitor capable of influencing TGF-β1 signaling pathways linked to fibrogenic responses.

## Discussion

In this study, we carried out an SBVS campaign to identify potential CDK8 inhibitors and successfully discovered a novel CDK8 inhibitor, P162-0948. The human kinome consists of 518 members; however, roughly two dozen members have FDA-approved drugs.^32^ While there exist potent CDK8 inhibitors, such as senexin A,^13^ cortistatin A,^44^ and CCT251921,^13,45^ no CDK8 inhibitors have gained U.S. FDA approval at the time of writing. Several explanations for this include low kinase selectivity, high toxicity, the development of drug resistance, poor bioavailability, etc. The binding site shared across the human kinome is relatively conserved, making kinase selectivity difficult.^17,24^ Identifying additional CDK8 inhibitors would better inform future drug design studies.

Molecular docking shows that P162-0948 favorably occupies the CDK8 binding site (Figure 2). Hydrogen bonds to the hinge residues are significant characteristics of kinase inhibitors.^24,32^ However, reports suggest that typical small-molecule kinase inhibitors will form one to three hydrogen bonds to the hinge residues, suggesting that more interactions of this type do not equate to potency.^24^ P162-0948 generated a hydrogen bond to hinge residue A100 (Figure 2). Due to the conserved nature of the kinase binding site, minute differences can be exploited to increase inhibitor selectivity. The catalytic residue K52 forms a salt bridge to residue E66, thereby stabilizing the kinase in the active conformation.^13^ The generation of a hydrogen bond by P162-0948 could disrupt the formation of the salt bridge, thereby inducing an inactive CDK8 conformation.^13,34^ Previously, we reported the identification of CDK8 inhibitors using the CDK8 DMG-in conformation.^46,47^ The CDK8 structure in this study contains a DMG loop (typically DFG in kinases) in the out conformation, revealing a large hydrophobic back pocket (Figure 2). This would suggest that the model would have a preference for small molecules with Type II structural features.^27,32^

The selected molecules with low CDK8 inhibitory activity lack favorable interactions observed with Type II kinase inhibitors (Figure 3). While P162-0948 is able to extend into the hydrophobic back pocket, comparisons to Type II CDK8 inhibitors suggest that additional optimizations can be performed to increase potency (Figure S2). For example, previous studies have utilized chloride halogens or morpholine moieties to take advantage of the hydrophobic back pocket.^34,36^ Previous pharmacological interactions considered the CDK8 DMG-in conformation.^46^ Molecules targeting the CDK8 DMG-out conformation contains a preference for hydrogen bonds to residues E66 and D173 and several residues located within the hydrophobic back pocket, such as L69, V147, and M174. This was evident when analyzing CDK8 Type II cocrystal ligands (Figure S3A,B). Considering the target protein structure is important as pharmacological interactions can impact the final screening selections (Figure S3C). Future optimization studies of P162-0948 could utilize bulky hydrophobic moieties, which may increase potencies toward CDK8 (Figure S2C).

CDK8 offers an interesting target for many diseases due to its involvement in the transcription of inflammatory signaling. To determine how well CDK8 would function as a therapeutic target, effective small-molecule inhibitors will need to be developed. We found P162-0948 to be not only potent (IC_50_ 50.4 nM) but also selective toward CDK8 (Figure 4). Both Tanimoto similarity scores and t-SNE calculations were performed to determine the structural novelty of P162-0948 (Figure 5). Tanimoto similarity is an important metric to measure molecular similarity; however, its calculations can lose information due to averaging distances over all structural fingerprint bits.^39,40^ In contrast, t-SNE seeks to preserve data structures from high-dimensional data.^40^ A molecule with a low Tanimoto similarity score was found to be placed near P162-0948 (Figure 5B,C). Both molecules contain an extended ring system but contain different substructures, which could influence their relationship. Taken together, P162-0948 is structurally distinct from known CDK8 inhibitors. Taken together, P162-0948 could serve as a starting point for additional design and optimization studies for the development of potent and selective CDK8 probes.

Current treatments for IPF include pirfenidone and nintedanib, both of which exert anti-inflammatory effects; however, they only slow damage to the pulmonary tissues.^5,6^ Additionally, their long-term use can cause discomfort, which reduces their effectiveness in some patients.^5,6^ CDK8 is an alternative therapeutic target due to its role in modulating the TGF-β/Smad axis. TGF-β remains a crucial cytokine that induces EMT and promotes epithelial cell migration.^10^ CDK8 has been shown to phosphorylate Smad 3 at T179 and RNA polymerase II, thereby regulating TGF-β-induced EMT.^15,16^ Our results show that P162-0948 can suppress cell migration in A549 cell cultures (Figure 6). Cell migration is crucial in the EMT process. TGF-β can trigger EMT, resulting in the fibrosis of the lungs.^10,14^ Research has shown that proteins involved in the TGF-β/Smad axis can be targeted by small molecules and disrupt pulmonary inflammation and lung fibrosis.^15,48^ TGF-β induced expression of EMT-related proteins, such as COL1A1, α-SMA, and Snail can be reduced when cotreated with P162-0948 (Figure 7). CDK8 can also form a transcriptional complex in the nucleus.^10,14^ This can also impact the phosphorylation of Smad, which indicates CDK8 as an alternative therapeutic target alongside the TGF-β1/Smad3 signaling pathway.^10,14^ This was confirmed in A549 cells treated with P162-0948 and TGF-β. Nuclear expression of CDK8 was not impacted, yet proteins associated with the TGF-β/Smad signaling axis showed reduced expression when treated with P162-0948 (Figure 8). These results would confirm previous reports of Smad3 phosphorylation at T179.^3,15,49^

To conclude, we identified P162-0948 as a novel CDK8 inhibitor through an SBVS campaign. Its CDK8 inhibitory activity was confirmed with enzymatic kinase assays. Cellular assays further identified its effectiveness in inhibiting CDK8 activity and demonstrated how it can mediate cell migration and EMT protein expression. These results serve as important starting points for the future development of potent and selective CDK8 inhibitors for therapeutic studies targeting fibrosis.

## Data Availability

The cocrystal structure (PDB ID: 4F6U) used for virtual screening was downloaded from the Protein Data Bank.^19^ The screening library was downloaded from ChemDiv (https://www.chemdiv.com). Information associated with this report is available online at the following GitHub repository (https://github.com/TonyEightLin/cdk8_virtual_screen.git). Molecular docking was performed using the Schrödinger Maestro software suite.^18^ Docking poses of compounds were visualized using PyMol. Docking poses of the protein and ligands can be found on the referenced repository as.pdb and.sdf files, respectively. Information from Table 1 can be found as a .csv file. The results of the similarity matrix and scatterplot are available as separate .csv files. Molecules from ChEMBL with CDK8 bioactivity are also available in .csv format. The IC_50_ data is available in .csv format. The curve was created using the Python package py50 (https://github.com/tlint101/py50.git).

## Supporting Information Available

Additional figures confirming molecular docking protocol and structural characteristics of CDK8 and Type II inhibitors (cocrystal structure selection, structural characteristics of P162-0948 and Type II inhibitors, and interactions between Type I and Type II CDK8 inhibitors) (PDF)
