# Supplementary material for: Identification of a Potent CDK8 Inhibitor Using Structure-Based Virtual Screening
Source: J Chem Inf Model. 2024 Dec 31;65(1):378–89. doi: 10.1021/acs.jcim.4c02011 (PMC11733953; doi:10.1021/acs.jcim.4c02011)
Supplement: Supplementary file 1 — ci4c02011_si_001.pdf [file ci4c02011_si_001.pdf]

Identification of a Potent CDK8 Inhibitor Using Structure-Based Virtual Screening

Tony Eight Lin[1,2, #], Ching-Hsuan Chou[3,#], Yi-Wen Wu[1], Tzu-Ying Sung[1], Jui-Yi Hsu[1,2], Shih-Chung Yen[4], Jui-Hua Hsieh[5], Yu-Wei Chang[2,6], Shiow-Lin Pan[1,2,7,8], Wei-Jan Huang[7,9] Kai-Cheng Hsu[1,2,7,8,10,*], Chia-Ron Yang[3,*]

[1]Graduate Institute of Cancer Biology and Drug Discovery, College of Medical Science and Technology, Taipei Medical University, Taipei, Taiwan

[2]Ph.D. Program for Cancer Molecular Biology and Drug Discovery, College of Medical Science and Technology, Taipei Medical University, Taipei, Taiwan

[3]School of Pharmacy, College of Medicine, National Taiwan University, Taipei, Taiwan

[4]Warshel Institute for Computational Biology, The Chinese University of Hong Kong (Shenzhen), Shenzhen, Guangdong, People's Republic of China

[5]Division of Translational Toxicology, National Institute of Environmental Health Sciences, National Institutes of Health, Durham, NC, USA

[6]Department of Traditional Chinese Medicine, Chang Gung Memorial Hospital, Keelung Medical Center, Keelung, Taiwan

[7]Ph.D. Program in Drug Discovery and Development Industry, College of Pharmacy, Taipei Medical University, Taipei, Taiwan

[8]TMU Research Center of Cancer Translational Medicine, Taipei Medical University, Taipei, Taiwan

[9]School of Pharmacy, Taipei Medical University, Taipei, Taiwan

[10]Cancer Center, Wan Fang Hospital, Taipei Medical University, Taipei, Taiwan

[#]These authors contributed equally to this work

Corresponding authors:
Kai-Cheng Hsu
E-mail: piki@tmu.edu.tw (K.C. Hsu)
TEL.: 886-2-66202589 #11106

Chia-Ron Yang
E-mail: cryang@ntu.edu.tw (C.R. Yang)
TEL.: 886-2-33668758

A

Type	PDB	Ligand	Resolution (Å)	DFG
Type II	4F6U	0SR	2.1	out
Type II	3RGF	BAX	2.2	out
Type II	4F6W	0SS	2.39	out
Type II	5HVY	66X	2.39	out
Type II	4F6S	0SQ	2.6	out
Type II	4F7J	0SU	2.6	out
Type II	4F7N	0SV	2.65	out
Type II	6TPA	NZ8	2.8	out
Type II	4F7L	0SO	2.9	out
Type II	4F70	0ST	3.0	out
Type I	5XS2	8D6	2.04	in
Type I	5ICP	69Z	2.18	in
Type I	6R3S	JRE	2.19	in
Type I	4F7S	0SW	2.2	in
Type I	5CEI	50R	2.24	in
Type I	5IDN	6A7	2.26	in
Type I	5XQX	8CC	2.3	in
Type I	5HNB	62M	2.35	in
Type I	5FGK	5XG	2.36	in
Type I	5HBE	5Y6	2.38	in
Type I	4CRL	C1I	2.4	in
Type I	6T41	MFE	2.45	in
Type I	6QTJ	JHK	2.48	in
Type I	5HBH	5Y7	2.5	in
Type I	5I5Z	68U	2.6	in
Type I	5BNJ	4TV	2.64	in
Type I	5IDP	6A6	2.65	in
Type I	6QTG	JH8	2.7	in
Type I	5HBJ	5Y8	3.0	in

B

RMSD (Å)	Cross docking			
	4F6U	3RGF	4F6W	5HVY
0SR	0.33	12.28	0.74	8.82
BAX	1.37	0.42	2.24	1.39
0SS	2.17	9.98	0.74	10.1
66X	1.6	2.66	11.32	1.29

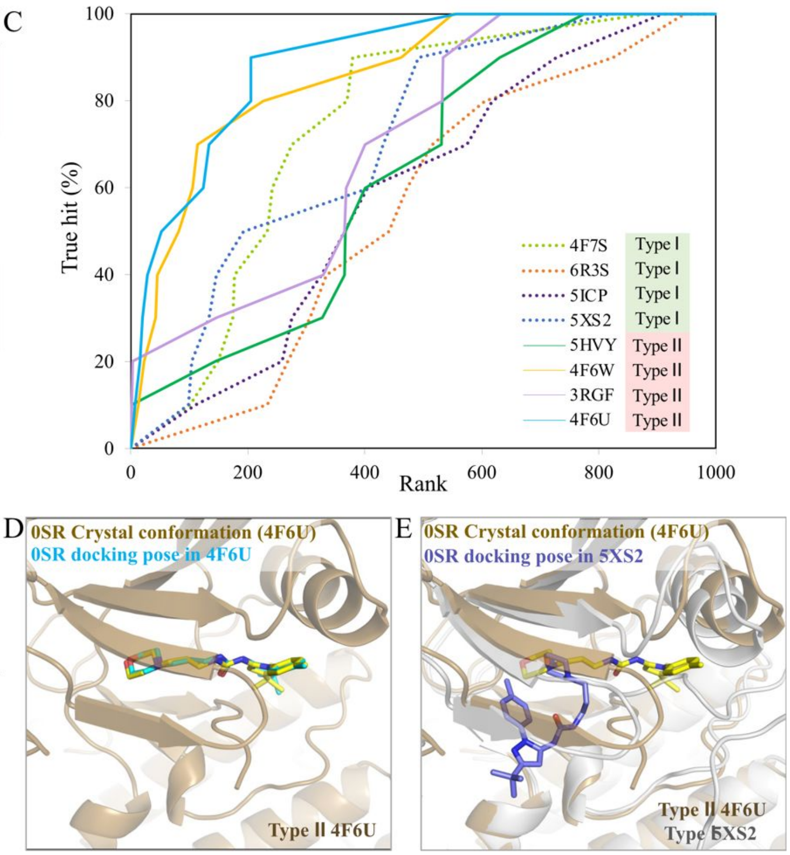

Figure S1. Co-crystal structure selection. Several steps were performed to determine the optimal structure for the screening protocol. (A) All CDK8 co-crystal structures were sorted by DMG position and resolution. The top four structures were selected for additional analysis. (B) Cross docking of the top four DMG-out structures was performed using their respective co-crystal ligand. The performance was measured based on the RMSD results of the final docking pose. (C) Docking performance was assessed by mixing the co-crystal ligands with 990 randomly selected molecules from the Available Chemical Directory (ACD). CDK8 structures with the DMG-out conformation show greater performance for discriminating Type II inhibitors. (D) The docking pose of 4F6U (blue) shows the most similar conformation to its co-crystal structure (yellow). (E) Superimposing 4F6U (brown, Type II) with 5XS2 (gray, Type I) shows structural differences that impede entrance to the hydrophobic back pocket. Docking the co-crystal ligand (purple) shows Type II inhibitors cannot access the hydrophobic back pocket.

A

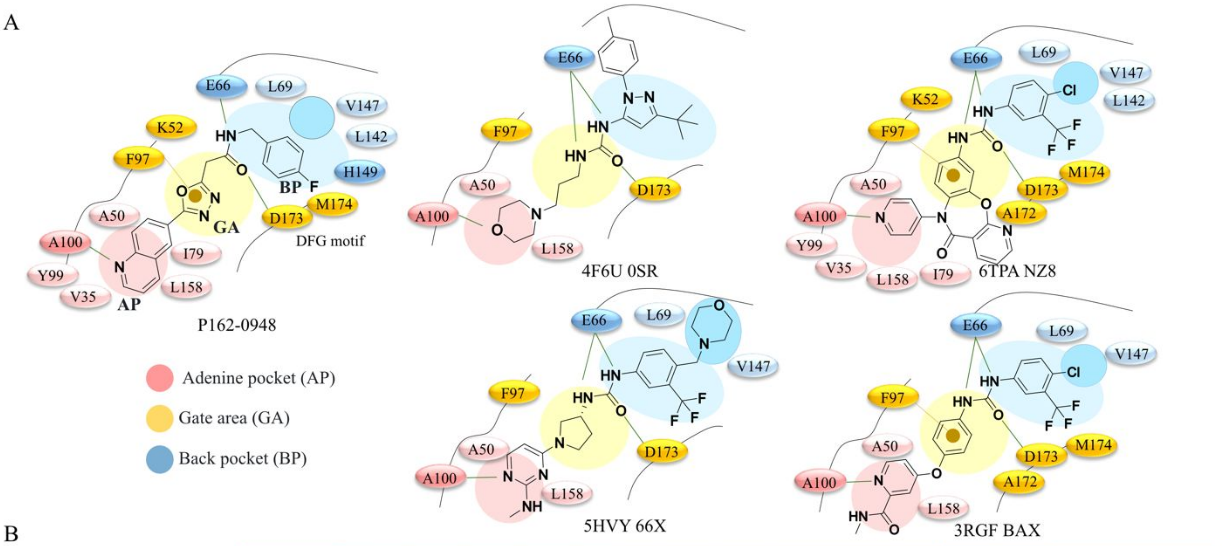

B

PDB	Ligand	Inhibition	AP	AP	Adjacent AP	Adjacent AP	Adjacent AP	Adjacent AP	Adjacent AP	GA	GA	GA	GA	GA	GA	BP	BP	Adjacent BP	Adjacent BP	Adjacent BP
			H	V	V	V	V	V	V	H	H	V	V	V	V	H	V	V	V	V
			A100	A100	V35	A50	I79	L158	Y99	K52	D173	K52	F97	A172	M174	E66	H149	L69	L142	V147
4F6U	P162-0948	IC_{50} : 50.4 nM	1	1	2	2	2	2	1		1	1	1 (Aromatic)		1	1	1			
4F6U	0SR	Kd : 700 nM	1	1		1		1			1		1 (chain)			1				
6TPA	NZ8	IC_{50} : 36.6 nM	1	1	1	1	1	1	1	1	1		1 (Aromatic)	1	2	2		1	2	1
5HVY	66X	IC_{50} : 17.4 nM	1	1		1		1			1		1 (Ring)					1		1
3RGF	BAX	IC_{50}: 8.25 nM	1	1		1		1			1	1	1 (Aromatic)	1	1	2		1	1	1

C

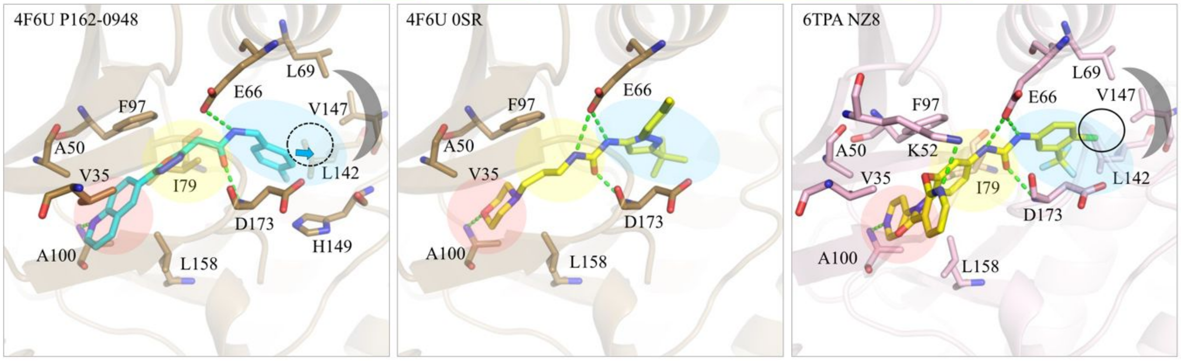

Figure S2. Structural characteristics of P162-0948 and Type II inhibitors. P162-0948 was compared to known Type II CDK8 inhibitors and structures from PDB. (A) Type II inhibitors occupy the adenine pocket (red), gate area (yellow), and a hydrophobic back pocket (blue). (B) Interaction table of known for Type II inhibitors. Red boxes highlight interactions generated by P162-0948. Blue box highlights interactions not found with P162-0948. (C) A secondary back pocket could potentially lead to more potent CDK8 inhibition.

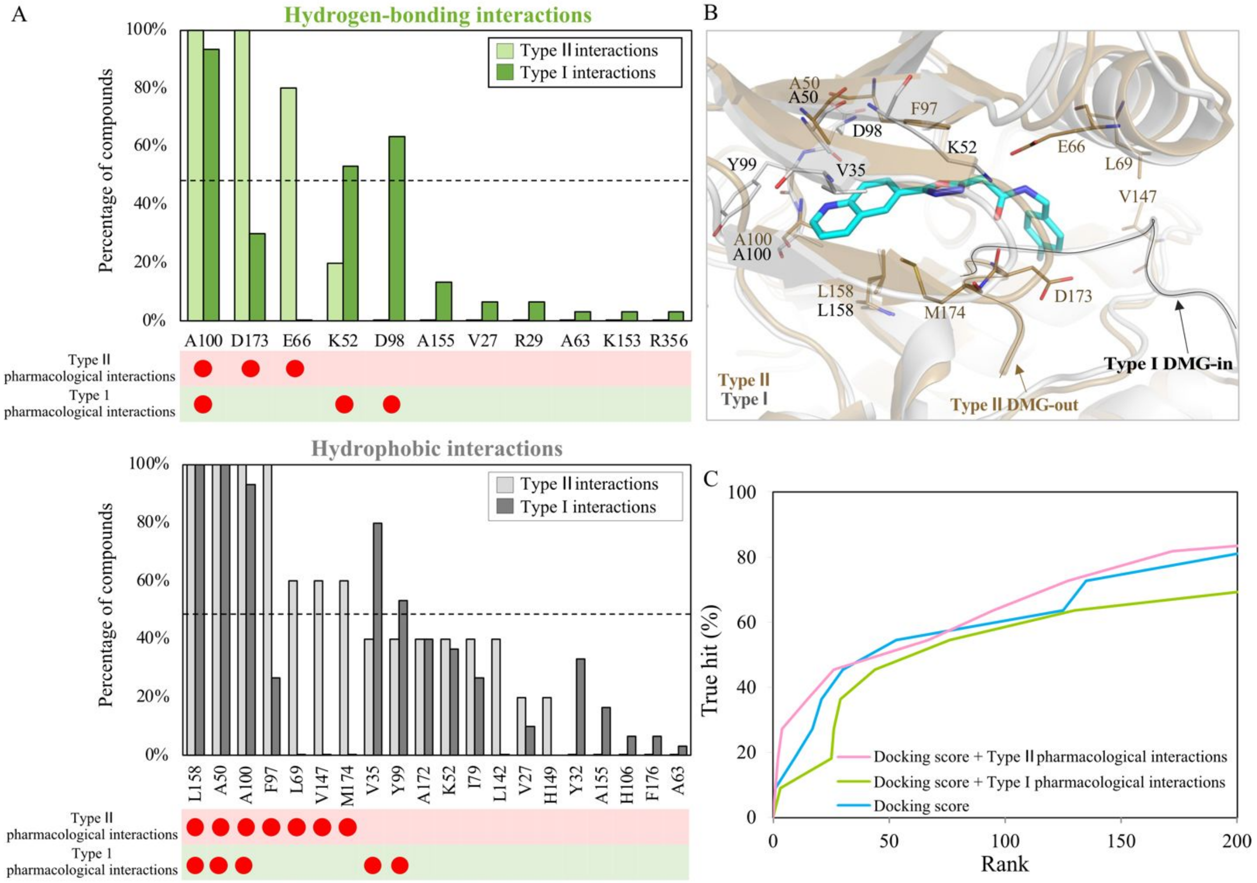

Figure S3. Interactions between Type I and Type II CDK8 inhibitors. Pharmacological interactions were analyzed using Type II co-crystal structures in **Figure S1A**. (A) A hydrogen bond to E66 and hydrophobic interactions with back pocket residues occurred more frequently with Type II inhibitors. (B) The binding pose of P162-0948 (blue) in the CDK8 structure 4F6U, which contains the DMG-out conformation. The CDK8 DMG-in conformation was superimposed, and the DMG loop is highlighted in black. (C) The Type II inhibitors were mixed with 990 ACD molecules, docked, and then ranked based on their docking score and pharmacological interactions. Consideration of Type II pharmacological interactions showed better performance with the CDK8 DMG-out conformation.
